# Mindfulness-Based Stress Reduction for Coronary Artery Disease Patients: Potential Improvements in Mastery and Depressive Symptoms

**DOI:** 10.1007/s10880-021-09822-z

**Published:** 2021-09-28

**Authors:** Oskar Lundgren, Peter Garvin, Lennart Nilsson, Viktor Tornerefelt, Gerhard Andersson, Margareta Kristenson, Lena Jonasson

**Affiliations:** 1 Crown Princess Victoria Children’s Hospital, Linköping, Sweden; 2 Division of Community Medicine, Department of Medical and Health Sciences, Linköping University, SE-581 83 Linköping, Sweden; 3 Research and Development Unit, and Department of Medical and Health Sciences, Linköping University, 581 85 Linköping, Sweden; 4 Division of Cardiovascular Medicine, Department of Medical and Health Sciences, Linköping University, 581 83 Linköping, Sweden; 5 Department of Behavioural Sciences and Learning, Linköping University, 581 83 Linköping, Sweden; 6 Department of Clinical Neuroscience, Section of Psychiatry, Karolinska Institute, 171 77 Stockholm, Sweden

**Keywords:** Coronary artery disease, Myocardial infarction, Mastery, Depression, Mindfulness

## Abstract

Depressive symptoms after coronary events are associated with a worse prognosis. When changing the focus from psychopathology towards a resilience framework, treatments such as mindfulness meditation could offer novel ways to address psychological distress among coronary artery disease (CAD) patients. We studied the feasibility of mindfulness-based stress reduction (MBSR) for CAD patients with depressive symptoms. Seventy-nine CAD patients with elevated depressive symptoms were invited to an 8-week MBSR course. Twenty-four patients (30%) accepted and 16 (20%) completed MBSR. Depressive symptoms decreased immediately after the course (*p* = .006). After 12 months, this improvement remained, and Mastery scores increased (*p* = .005). A reference group of 108 CAD patients did not show any significant changes in depressive symptoms or Mastery between 1 and 12 months after a coronary event. MBSR thus appears to be a feasible alternative for CAD patients with elevated depressive symptoms. Future studies are warranted to study if MBSR can improve psychological functioning in CAD patients.

Clinicaltrials.gov (Registration Number: NCT03340948).

## Introduction

Depressive symptoms are common among patients with coronary artery disease (CAD) and are associated with a worse prognosis (Lichtman et al., 2008; Sher et al., 2010). Almost one out of five CAD patients are clinically depressed (Meijer et al., 2011). Depression following acute myocardial infarction is also associated with adverse outcomes, such as all-cause mortality, cardiac mortality, and new cardiovascular events (Meijer et al., 2011). Randomized controlled trials of pharmacological therapy after myocardial infarction have shown small to moderate effects on depression status and no reduction in coronary events (Berkman et al., 2003; Glassman et al., 2009; van Melle et al., 2007). A recent longitudinal study even demonstrated a worse prognosis among those who responded with reductions in depressive symptoms (Grace et al., 2018). Psychological treatments have been tested (e.g., cognitive therapy) and show small to moderate improvement in depression and a small effect on cardiac mortality. However, heterogeneity in findings and unclear methodologies have prevented large-scale implementation of psychological treatments (Whalley et al., 2014).

The narrow focus on treating psychopathology has recently been criticized for limiting the development of new interventions (Kalisch et al., 2017). A focus on resilience and coping resources has been proposed to create new possibilities for developing of treatments and preventive efforts to intervene against stress-related disorders. Having a serious illness is, for most people, a very stressful experience. According to the cognitive activation theory of stress, coping is defined as expecting positive outcomes based on earlier experience of adaptive responses to stressful situations (Ursin & Eriksen, 2004). This sense of ability to handle life’s hardships, also termed mastery, contains both feelings of confidence and self-reliance (Pearlin & Schooler, 1978). Maladaptive coping leads to negative expectations and low confidence, and if prolonged, may result in states such as hopelessness (Orwilius et al., 2017), depression, and exhaustion (Ursin & Eriksen, 2004).

Compared to psychological risk factors, less attention has been paid to the role of psychological resources and CAD. However, a few longitudinal population-based studies have shown protective effects of Mastery (Surtees et al., 2006), Self-esteem (Stamatakis et al., 2004), Life enjoyment (Shirai et al., 2009), and Emotional vitality Kubzansky & Thurston, 2007) on CAD incidence. Moreover, Mastery significantly and independently reduced the 8-year risk of CAD in a normal population study (Lundgren et al., 2015). A high sense of Mastery could be an essential asset for patients who are doing their best to cope with a new reality, such as living with a diagnosis of CAD.

Based on what is known about psychological risk factors and resources and their influence on CAD risk, there is an unmet need for new psychological interventions in cardiac rehabilitation. Emerging psychological treatments, grounded in a resource perspective, are generally targeting psychological processes upstream of distressful psychological contents (e.g., thoughts and feelings). Thus, they aim to change how patients relate to their experiences, how they view their lives, and how they respond to challenges and opportunities (Kalisch et al., 2017; Tang et al., 2015).

With rising popularity in medical contexts, Mindfulness-Based Stress Reduction (MBSR) could be a suitable group intervention for distressed CAD patients. Jon Kabat-Zinn et al. developed the 8-week MBSR-course in the 1980s (Kabat-Zinn et al., 1985). It consists of 40 min of daily mindfulness meditation or yoga practice 6 days a week. The mindfulness meditation practices are carried out in sitting, standing, or lying down positions, in which the participants are practicing either one-pointed concentrated attention or paying attention with a more open and fluid awareness that monitors the constant flow of experiences (Kabat-Zinn, 2013). The central theme of mindfulness is defined as paying attention, on purpose, in the present moment, and as non-judgmentally as possible. Researchers are currently gaining a growing understanding of how this kind of mental training influences the human brain and downstream biological systems (Tang et al., 2015).

Loucks et al. (2015) reported a positive association between dispositional mindfulness (a self-reported trait) and cardiovascular health in a cross-sectional population-based study. Moreover, the positive effects of mindfulness training on depressive symptoms in chronic diseases (Goyal et al., 2014) and psychiatric disorders (Goldberg et al., 2018) have been documented. Interestingly, two earlier studies of modified MBSR courses, including mindfulness-based cognitive therapy (O’Doherty et al., 2015) and a brief 3-week MBSR-inspired intervention (Nyklíček et al., 2015), reported improvements in depression and anxiety in CAD patients. Also, MBSR has shown positive effects on depressive symptoms and on heart failure symptoms, including tiredness, in patients with known heart failure (Norman et al., 2018; Sullivan et al., 2009). In summary, mindfulness seems to be a good candidate treatment that rests on a foundational resilience framework to reduce stress and distress among patients with severe and chronic diseases.

This study aimed to investigate the feasibility and acceptability of the 8-week MBSR course among CAD patients who showed depressive symptoms after a recent coronary event. We also measured effects on Mastery and changes in sleep quality and levels of anxiety, up to 12 months after the course. A second aim was to compare the temporal patterns of outcome measures in MBSR-participants with a larger observational cohort of CAD patients.

## Materials and Methods

### Intervention Group

During 2012–2013, CAD patients who were referred to the outpatient clinic of the Department of Cardiology, Linköping University Hospital, Sweden, were asked to fill out the Centre for Epidemiological Studies Depression scale (CES-D) questionnaire (Radloff, 1977) one month after their coronary event, i.e., myocardial infarction or coronary revascularization procedures; percutaneous coronary intervention (PCI) or coronary by-pass-graft surgery (CABG). Patients were then consecutively identified, and invitation letters for participation in MBSR were sent.

Based on the median value at one month in the observational cohort (the so-called reference group), CES-D scores > 8 was chosen to define those with elevated depressive symptoms in the intervention group. This cut-off was chosen to include patients with subclinical levels of depressive symptoms. Earlier studies have suggested that an arbitrary cut-off of 16 points would represent a clinical threshold (Radloff, 1977). Exclusion criteria were significant clinical depression (based on cardiologist’s clinical judgment), severe comorbidities, such as cancer, severe cognitive impairment, psychosis, and alcohol or drug abuse, since these conditions might imply difficulties with completing MBSR. Patients filling these criteria were then consecutively identified, and invitation letters for participation in MBSR were sent. This population is hereafter referred to as the intervention group.

**Fig. 1:**
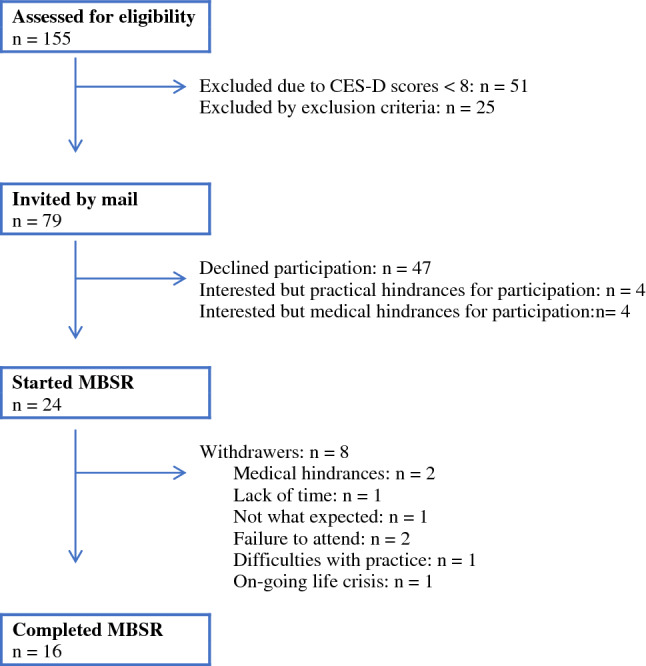
Flow-chart of recruitment and participation in the 8-week MBSR program

A flow chart of the recruitment to MBSR is shown in Fig. 1. In total, 155 patients were assessed for eligibility. Invitation letters were sent to 79 patients (with CES-D > 8 and no exclusion criteria). Fifty-five (70%) declined to participate or had practical/medical impediments for participation. Patients who accepted the invitations received additional information about MBSR via a phone call. Twenty-four patients started the 8-week MBSR program, and 16 (20%) completed it, thus representing a completion rate of 67% for those who began the program. All participants who dropped out of the study did so during the first 3 weeks of MBSR. We aimed to reach an active MBSR group of 25 participants, as previously recommended (Kabat-Zinn et al., 1985). The Ethical Review Board of Linköping gave its approval to the study (registration number: 2013/17/31), and the trial was retrospectively registered in clinicaltrials.gov (registration number: NCT03340948).

### Reference Group

In 2011, a longitudinal survey of Mastery and depressive symptoms in CAD patients was performed at the same outpatient clinic as the intervention group. In total, 179 consecutive patients filled out the CES-D questionnaire and the Mastery scale (Pearlin & Schooler, 1978) 1 month after discharge. Among them, 108 (72%) returned for a 12-month visit and filled out both questionnaires a second time. The purpose of this observational population, hereafter called the reference group, was to; (a) measure the levels and stability of Mastery and CES-D over the first year after a coronary event, and (b) based on CES-D scores at 1 month, define a cut-off level for elevated scores to be used in the recruitment of the intervention group.

### Mindfulness-Based Stress Reduction

The 8-week long MBSR program consisted of 8 weekly 2.5 h group sessions and one all-day (6 h) practice session in week 6 (Kabat-Zinn, 2013). The participants also received CDs with guided instructions and were recommended to practice for 40 min, 6 days a week. The central theme was a variety of mindfulness practices. Body scan was performed lying down, and attention was systematically moved through the body. Sitting meditation was practiced with either focused one-pointed attention (e.g., to the breath) or with open monitoring of the constantly changing flow of experience. Yoga sessions consisted of movement in and out of specific poses, with continuous awareness of bodily sensations. Participants were encouraged to keep a journal and write down reflections of their experiences after practice sessions. Moreover, the weekly meetings consisted of group dialogues about both on-going practice and topics related to stress biology and stress reduction. The only minor deviation from the MBSR manual was a 20-min dialogue about CAD and stress in session 4. The MBSR teacher was at the time of the study enrolled in the second phase of MBSR teacher training, had 3 years of teaching experience, and led the CAD patient group under supervision from a certified MBSR supervisor. A recent meta-analysis concluded that MBSR seems to be a safe intervention since reported adverse events have been rare in earlier studies (Wong et al., 2018).

### Psychological Assessments

Depressive symptoms were measured with the 20-item CES-D scale (Radloff, 1977), a self-report measure designed to assess depressive symptoms in the general population. The 9 item Patients Health Questionnaire-9 (PHQ-9), which corresponds to the depression criterion in the diagnostic manual DSM-4, was used to provide an additional mode of measurement of depressive symptoms in the intervention group (Kroenke & Spitzer, 2001). Mastery was measured with the Mastery scale developed by Pearlin and Schooler (1978). The 7-item instrument has shown good psychometric properties in Swedish samples of mentally ill and healthy populations (Eklund et al., 2012). Two psychological variables related to the proposed mechanisms involved in mindfulness meditation were also measured: Self-reported Mindfulness with the 29-item Five Facet Mindfulness Questionnaire (FFMQ) (Baer et al., 2008). Acceptance, conceptualized as low perceived psychological inflexibility (thus a low score is healthy), was measured with the 6-item AAQ-II questionnaire (Bond et al., 2011). Also, anxiety symptoms were measured with the 7-item Generalized Anxiety Scale-7 (GAD-7) (Spitzer et al., 2006). Sleep quality was measured with one item derived from the Karolinska Sleep Questionnaire (KSQ) (Kecklund & Åkerstedt, 1992). Global quality of life was measured with the 0–10 graded one-item scale Cantril’s Ladder of Life (Cantril, 1965).

### Participants’ Evaluation

The post-intervention questionnaire also contained ten visual analogue scale-items (VAS), graded 0–10, for evaluation of participants’ experience of MBSR and its specific components, e.g., weekly meetings and sitting meditation practice. Also, two categorical (yes/no) items addressed (1) whether participants would recommend MBSR to a friend and (2) if something of lasting value was learned (Table 3).

### Statistical Analysis

All variables showed normal distribution, by visibly plotting the distribution and computing a Shapiro–Wilk test, except for scale scores on Mindfulness and Mastery. Since most variables were normally distributed, data are presented as mean and standard deviation (SD). Since two variables deviated from the normal distribution and the study population was small, non-parametric analyses were used. Using Mann–Whitney *U* test, potential differences between groups were analysed. Changes over time were analysed by using the Wilcoxon sign rank test. A Chi-square test was used for nominal data. For test–retest correlations, we used Spearman correlation coefficients. In all analyses, a significance level of *p* < .05 was chosen to define statistical significance. All analyses were computed with IBM SPSS Version 24.

## Results

### MBSR and Feasibility in the Intervention Group

The baseline characteristics of the intervention group, divided according to whether they declined, accepted, or completed participation, are summarized in Table 1. Those who declined participation exhibited significantly lower CES-D scores compared with those who started the MBSR program. No other differences were seen between those who rejected and those who accepted, except for lower waist circumference levels in the former group. Earlier experience of mindfulness or yoga education was minimal among participants (1 out of 24 participants). 13 of the 16 completers attended 67% or more of the nine meetings. The average self-reported daily amount of practice was 21 min, representing 50% of the prescribed amount in the MBSR protocol. Participants’ evaluations of the MBSR program and its specific components are shown in Table 2. Overall, the patients reported a positive experience of MBSR, with yoga practice receiving the highest grade and reflective journaling the lowest. No adverse cardiac events (fatal- or non-fatal myocardial infarction or worsened angina) were observed in the MBSR group (neither completers nor withdrawers) during the 8-week course or 12-month follow-up.

**Table 1:** Baseline characteristics of patients with CES-D ≥ 8 who were invited to MBSR

	Declined	Accepted	Completed	*p*^a^
	*n* = 55	*n* = 24	*n* = 16	
Age, years	63 (8)	61 (9)	59 (10)	.096
Female, *n* (%)	18 (33)	9 (37)	5 (31)	.681
Waist circumference (cm)	96 (14)	105 (13)	105 (11)	.014
Smoking, *n* (%)	14 (26)	5 (22)	2 (12)	.697
Hypertension, *n* (%)	44 (80)	19 (80)	12 (75)	.932
Diabetes mellitus, *n* (%)	11 (20)	8 (33)	6 (38)	.202
Use of antidepressants^b^, *n* (%)	9 (17)	2 (8)	0 (0)	.134
*Index event*				
Myocardial infarction, *n* (%)	30 (55)	14 (58)	12 (75)	.755
PCI, *n* (%)	19 (34)	9 (38)	3 (19)	.801
CABG, *n* (%)	6 (11)	1 (4)	1 (6)	–
*CES-D scores*	15 (8)	19 (8)	20 (7)	.021

Values are given as mean (SD) if nothing else is indicated

Patients were divided into three subgroups; those who declined participation, those who accepted participation and those who completed the course

*PCI* Percutaneous coronary intervention, *CABG* Coronary artery by-pass graft surgery

^a^Differences between those who declined and those who accepted, calculated with Mann–Whitney test if means, and Chi-square test if proportions

^b^Prescription during the last 3 months

^c^Proportions too small to calculate significant difference

**Table 2:** Evaluation of participants’ experiences of the 8-week MBSR program assessed immediately after the intervention

Items	Mean grades 1–10 (10 = excellent)
Overall evaluation of the MBSR course	8.3
Weekly meetings at the hospital	8.1
Body-scan practice	7.4
Sitting meditation	7.4
Walking meditation	5.6
Yoga practice	8.0
Written reflections	5.4
Informal practice in everyday life	6.5
Teacher	9.0
Cours administration	9.1

Will recommend MBSR to a friend with health difficulties	100%
Learned something of lasting value	79%
Worth the time and energy	93%

### Psychological Variables in the Intervention Group

The modal value of months from the index event to the start of MBSR was 6 (range 3–11) in the 24 patients who started the course. Their CES-D scores immediately before the beginning did not differ from their scores 1 month after the coronary event (mean 20 *vs*. 19). Likewise, among those who completed MBSR (*n* = 16), CES-D scores before the start did not differ from their one-month scores (mean 19 *vs*. 20). The psychological assessments of CES-D, Mastery, Mindfulness, Acceptance, anxiety, sleep- and life quality in patients who completed the MBSR program (*n* = 16) are shown in Table 3. Among patients who completed the MBSR-program, mean scores in CES-D decreased from 20 to 13 (*p* = .03), and remained stable (mean 13) at 12 months (*p* = .005). Similarly, the PHQ-9 scores showed reductions in the same range at post-MBSR (mean 8.8 *vs*. 4.0, *p* = .002) and remained at this level at 12 months.

**Table 3:** Psychological variables in patients who completed the MBSR program (*n* = 16)

	1 m	Range	Pre-MBSR*	Post-MBSR	Pre- *vs* post-change (%)	Pre- *vs* post-*p*	12 m	Pre- *vs* 12 m change** (%)	Pre- *vs* 12 m *p*
CES-D	19 (7.0)	0–60	20 (8)	13 (8)	− 33	.006	13 (7)	− 33	.005
PHQ-9	–	0–27	8.8 (4)	4.9 (3.3)	− 44	.002	5.5 (3)	− 38	.012
Mastery	–	7–28	21 (2)	22 (3)	+ 6.3	.110	23 (3)	+ 8.7	.005
FFMQ	–	29–145	85 (9)	94 (12)	+ 11.5	.001	96 (12)	+ 14	.003
AAQ-II	–	7–49	19 (6)	15 (7)	− 20	.002	14 (5)	− 29	.002
GAD-7	–	0–21	7.5 (5)	4.3 (3)	− 43	.004	4.4 (2)	− 41	.005
KSQ	–	0–4	2.9 (1)	3.3 (1)	+ 14	.014	3.3 (1)	+ 14	.033
Ladder of life	–	0–10	5.6 (1)	6.5 (2)	+ 16	.034	6.5 (2)	+ 16	.038

Psychological variables assessed at the following occasions; 1 month after index event, immediately before MBSR (pre-MBSR), immediately after MBSR (post-MBSR), and 12 months after MBSR. Values are given as mean (SD)

Mean differences calculated with Wilcoxon sign rank test

*CES-D* Centre for Epidemiological Studies Depression, *PHQ-9* Patient Health Questionnaire-9, *FFMQ* Five Facet Mindfulness Questionnaire, *AAQ-II* Acceptance Action Questionnaire 2, *GAD* Generalized Anxiety Scale-7, *KSQ* Karolinska Sleep Questionnaire

*Mode number of months from index coronary event to start of MBSR; 6 (range 2–11 months)

**Relative change (%) in mean values of scores between the two occasions (difference/baseline)

We also observed an increase in Mastery scores over time that became significant after 12 months (mean scores, 21 *vs*. 22 *vs*. 23, the *p* value for pre-MBSR *vs*. 12 months was .005). In contrast, the eight patients who withdrew from the course (but still filled out the questionnaires) showed no changes in CES-D scores over the same period; before starting 19.0 (SD 12), after 8 weeks 20 (SD 7.0), and after 12 months 19 (SD 15) (data not shown in table). The number of valid Mastery questionnaires in the dropout group was too small (*n* = 3) to perform statistical analyses.

As shown in Table 3, the two variables related to the mindfulness practice showed significant improvements immediately after MBSR, and these effects were maintained after 12 months. Mean Mindfulness scores changed from 85 to 94, pre- *vs*. post-MBSR (*p* = .001), and was 96 at 12 months. Acceptance (i.e., experiential avoidance) scores changed from 19 to 15, pre- *vs*. post-MBSR (*p* = .002), and was 14 at 12 months. Anxiety scores changed from 7.5 to 4.3 pre- *vs*. post-MBSR (*p* = .004), and remained at 4.4 at 12 months. Mean scores at the sleep quality questionnaire increased from 2.9 to 3.3, pre- *vs*. post-MBSR (*p* = .014), and remained at 3.3 at 12 months. Quality of life, measured with the 10-step Ladder of Life, changed from 5.6 to 6.5, pre- *vs*. post-MBSR (*p* = .034), and remained at 6.5 after 12 months.

### Reference Group

The baseline characteristics of the reference group with data from both occasions are shown in Table 4. CES-D and Mastery scores are shown in Table 5. CES-D-scores did not change significantly between 1 and 12 months, neither in the whole reference group (mean 9.0 *vs*. 9.5, *p* = .886, nor among those with CES-D scores above the median (> 8) at 1 month (mean 14 *vs*. 12, *p* = .082). There were no significant changes in Mastery scores over time, neither in the whole reference group (mean 23 *vs*. 23, *p* = .144) nor in the subgroup with CES-D scores above the median (> 8) at 1 month (mean 22 *vs*. 22, *p* = .016). Test–retest correlations between 1 and 12 months were *r* = .55 for CES-D (*p* = .001) and *r* = .54 for Mastery (*p* = .001) respectively.

**Table 4:** Baseline characteristics of CAD patients in the reference group

	*n* = 108
Age, years, mean (SD)	63 (8.6)
Female, *n* (%)	28 (26)
Waist circumference, cm, mean (SD)	98 (13.5)
Smoking, *n* (%)	28 (26)
Hypertension, *n* (%)	58 (54)
Diabetes mellitus, *n* (%)	20 (19)
Use of antidepressants, *n* (%)^a^	6 (0.6)
*Index coronary event*	
Myocardial infarction, *n* (%)	72 (67)
Elective coronary intervention, *n* (%)^b^	36 (33)

^a^Self-reported intake during the last 3 months

^b^Patients who underwent PCI or CABG due to disabling angina pectoris

**Table 5:** Mean scores on the CES-D and Mastery scales in the reference group at 1 and 12 months after the index CAD event in all patients and in the subgroup with CES-D ≥ 8 at 1 month

	1 month	12 months	*p*
*CES-D*			
All patients, *n* = 108	9.0 (7.6)	9.5 (8.6)	.886
CES-D ≥ 8 at 1 month, *n* = 56	14 (7.2)	12 (8.1)	.082
*Mastery*			
All patients, *n* = 108	23 (3.0)	23 (3.1)	.144
Mastery (CES-D ≥ 8) at 1 month, *n* = 56	22 (2.7)	22 (2.9)	.116

Values are given as mean (SD)

## Discussion

This study suggests that MBSR can be a feasible treatment for a subgroup of CAD patients with persistent depressive symptoms after a coronary event. Moreover, the findings provide some initial evidence that MBSR could lead to significant and sustained improvements in both Mastery and depressive symptoms. Furthermore, the reference group showed that Mastery and depressive symptoms remained stable between 1 and 12 months after a coronary event.

These findings are in line with the hypothesis that mindfulness can be used in cardiac rehabilitation and adds to earlier trials with modified MBSR courses. To our knowledge, the present feasibility study is the first to test the acceptability of the full 8-week MBSR-program in this particular context.

Overall, the MBSR course was well-received and deemed acceptable among those who completed the program. Somewhat unexpectedly, those who accepted to participate exhibited higher CES-D scores than those who declined participation, suggesting that patients with higher levels of depressive symptoms were more motivated to engage in mindfulness training. Participants’ CES-D levels did not change between the first assessment (1 month after the CAD event) and the start of MBSR, indicating an absence of spontaneous improvement. 8 of 24 patients who started the MBSR program chose to withdraw from it. This could be interpreted as a negative outcome but also illustrates the well-known fact that participation in MBSR, paradoxically, can be stressful and demanding (Baer et al., 2012), especially for patients who show a lack of energy and vitality. However, our study’s completion rate (67%) was higher than that of O’Doherty et al. (2015). They reported a 53% completion rate among CAD patients referred to mindfulness-based cognitive therapy. In comparison with other disease groups, the completion rate in the present study was higher than in patients with irritable bowel syndrome (66%) (Zernicke et al., 2013) but lower than in an asthma population (85%) (Pbert et al., 2012).

The self-reported practice time in our study corresponds to about half the recommended amount. This is raising the question of whether this was sufficient or if more training is needed, which may require intensified motivational work. A modified version of the 8-week MBSR program, given to cancer survivors, a positive impact on fatigue, depression, and sleep disturbance was seen (Johns et al., 2015). In that study, as well as in the study by O’Doherty et al. (2015), the recommended daily home practice time was 20 min, thus resembling the amount of invested time by the participants in our study. Whether invested time and effort correlate with sustainable improvements in function and well-being merits further investigation. The related questions of intervention completion and adherence to practice recommendations connect to broader clinical issues in cardiac rehabilitation. Even though evidence is strong for many secondary preventive strategies, including physical activity and risk-lowering pharmacological treatments, most patients do not reach the goals of cardiac rehabilitation programmes (Kotseva et al., 2016). Whether improvements in psychological functioning also translates into increased motivation for change and improved adherence to treatment recommendations is a crucial future research question.

Although the study was designed as a feasibility trial, we also measured the effects of MBSR on depressive symptoms and Mastery. The significant and sustained reduction in depressive symptoms was expected. A systematic review has shown that mindfulness reliably improves depressive symptoms in patients with various chronic diseases, although studies on CAD patients are few (Goyal et al., 2014). Of note, the mean CES-D scores in our reference group did not change during the 12-month follow-up. This is in accordance with earlier studies, which have shown that depressive symptoms remain unchanged during the first year after a coronary event (Paraschar et al., 2006; Thombs et al., 2006). Our finding that the second measure of depressive symptoms (PHQ-9) was influenced in the same direction and to a similar degree provides concurrent evidence for the validity of using CES-D as both a screening tool and outcome measure when evaluating the effects of MBSR in this particular context.

The significant increase in Mastery, which was observed at 12 months after the MBSR course, was a less expected finding. To our knowledge, this is the first study that follows Mastery scores prospectively in patients with a recent coronary event. The late onset of improvement may reflect that Mastery is a more stable trait that requires more than 8 weeks to reveal a change. Indeed, Pearlin (2010), who developed the Mastery scale, describes the concept as learned appraisals of coping capabilities concerning life circumstances that one has to face. Thus, one reason behind the delayed improvement may be that the participants’ newly developed coping skills were needed to be tested in real life, with its inevitable hardships. The overall pattern of reductions in anxiety and experiential avoidance (acceptance), and increases in sleep quality and quality of life may provide indirect evidence that the patients’ lives were experienced as more manageable after participating in MBSR*.* Furthermore, the findings of delayed increases in Mastery highlight the importance of extended follow-up after psychosocial interventions since significant long-term effects might be missed otherwise.

Some limitations need to be mentioned. The small number of participants in the intervention group does not allow us to draw firm conclusions of effectiveness. Moreover, variations in time from diagnosis to start of MBSR in the intervention group prevents statistical analyses of differences with the reference group. While our study’s pre-post design is suitable for the investigation of feasibility, it lacks the benefits of randomization to active intervention or active control group when addressing effectiveness.

### Conclusions

We conclude that MBSR can be a feasible and acceptable alternative for CAD patients with persistent depressive symptoms after a coronary event. MBSR was also associated with sustained improvements in both Mastery and depressive symptoms. However, more studies are needed to determine the role of MBSR as a complementary treatment in CAD patients.

## Data Availability

The dataset analysed during the current study are available from the corresponding author on reasonable request.
